# The Surviving, Not Thriving, Photoreceptors in Patients with *ABCA4* Stargardt Disease

**DOI:** 10.3390/diagnostics14141545

**Published:** 2024-07-17

**Authors:** Hanna De Bruyn, Megan Johnson, Madelyn Moretti, Saleh Ahmed, Mircea Mujat, James D. Akula, Tomislav Glavan, Ivana Mihalek, Sigrid Aslaksen, Laurie L. Molday, Robert S. Molday, Bruce A. Berkowitz, Anne B. Fulton

**Affiliations:** 1 Department of Ophthalmology, Boston Children’s Hospital, Boston, MA 02115, USA; hanna.debruyn@childrens.harvard.edu (H.D.B.);; 2 Department of Ophthalmology, Visual and Anatomical Sciences, Wayne State University School of Medicine, Detroit, MI 48201, USA; 3 Physical Sciences, Inc., 20 New England Business Center, Andover, MA 01810, USA; mujat@psicorp.com; 4 Department of Ophthalmology, Harvard Medical School, Boston, MA 02115, USA; 5 Department of Molecular Medicine and Biotechnology, Faculty of Medicine, University of Rijeka, 51000 Rijeka, Croatia; 6 Department of Biochemistry and Molecular Biology, University of British Columbia, Vancouver, BC V6T 1Z4, Canada; 7 Department of Clinical Science, University of Bergen, 5007 Bergen, Norway; 8 Department of Medical Genetics, Haukeland University Hospital, 5009 Bergen, Norway

**Keywords:** Stargardt disease, photoreceptors, mitochondria, optical coherence tomography adaptive optics, inherited retinal disease, retinal imaging, retina

## Abstract

Stargardt disease (STGD1), associated with biallelic variants in the *ABCA4* gene, is the most common heritable macular dystrophy and is currently untreatable. To identify potential treatment targets, we characterized surviving STGD1 photoreceptors. We used clinical data to identify macular regions with surviving STGD1 photoreceptors. We compared the hyperreflective bands in the optical coherence tomographic (OCT) images that correspond to structures in the STGD1 photoreceptor inner segments to those in controls. We used adaptive optics scanning light ophthalmoscopy (AO-SLO) to study the distribution of cones and AO-OCT to evaluate the interface of photoreceptors and retinal pigment epithelium (RPE). We found that the profile of the hyperreflective bands differed dramatically between patients with STGD1 and controls. AO-SLOs showed patches in which cone densities were similar to those in healthy retinas and others in which the cone population was sparse. In regions replete with cones, there was no debris at the photoreceptor-RPE interface. In regions with sparse cones, there was abundant debris. Our results raise the possibility that pharmaceutical means may protect surviving photoreceptors and so mitigate vision loss in patients with STGD1.

## 1. Introduction

Stargardt disease (STGD1), due to biallelic variants in the *ABCA4* gene, is the most common juvenile macular dystrophy, although late-onset forms are recognized [1,2,3,4,5,6,7,8,9,10]. Both early and late forms cause similar changes in retinal structure and function, devastate vision, and are currently untreatable. The natural course of STGD1 leads to the gradual demise of photoreceptors and retinal pigment epithelial (RPE) cells. Retinal imaging is an integral part of clinical diagnosis, even in this era of accessible molecular genetics. In this paper, we focus on retinal structure and, where possible, deduce the molecular implications of structure delineated by the retinal imaging. First, we introduce the molecular and structural underpinnings of the disease and the clinically recognizable severe and more mild forms of STGD1 photoreceptor disease. This is followed by the presentation and analysis of biomarkers represented in spectral domain optical coherence tomographic (SD-OCT) images of the photoreceptors and in adaptive optics (AO) scanning light ophthalmoscope (SLO) and OCT images of STGD1 photoreceptors.

**Figure 1:**
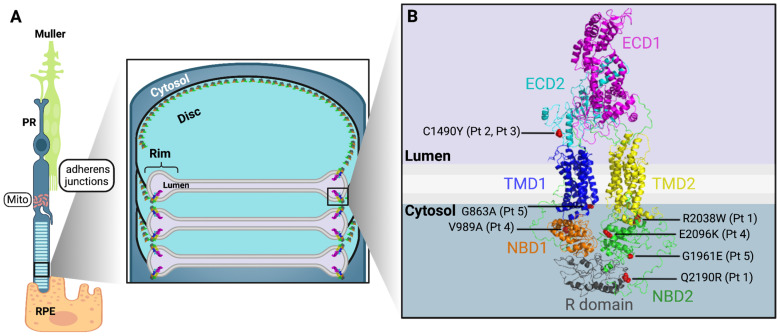
Photoreceptor (PR), retinal pigment epithelium (RPE), and *ABCA4* protein. (**A**) The PR and RPE have close structural and functional relationships. *ABCA4* protein is located in the rim of the discs of the photoreceptor outer segment. In the photoreceptor’s inner segment is the ellipsoid zone (EZ), which has abundant mitochondria (Mito), which are needed to support the high energy demands of the photoreceptor. Also, at the level of the PR inner segment, adherens junctions form the external limiting membrane (ELM), one of the OCT hyperreflective bands. Images were adapted from Scortecci et al. [14] and Steinberg et al. [15]. (**B**) This diagram of *ABCA4* protein highlights its functional domains and indicates the site of variants found in our patients. The extracellular domains 1 and 2 (ECD1, ECD2) reside in the lumen, while the transmembrane domains 1 and 2 (TMD1, TMD2) are embedded in the lipid bilayer of the disc. The nucleotide-binding domains 1 and 2 (NBD1, NBD2) are in the cytosol [16]. In Patient 1, R2038W and Q2190R are on the same allele; on the other allele, there is a deep intronic variant resulting in complete protein loss. Patient 2 and Patient 3 have C1490Y on one allele; their other allele contains a deep intronic variant. In Patient 4, the two variants are V989A and E2096K, and in Patient 5, the two variants are G863A and G1961E, which are located as indicated. See Table 1, as well. For details about variants and genotypes, please see Appendix A.

Normally, *ABCA4* protein, an ATP-powered flippase located in the rim of the photoreceptor disk (Figure 1A), clears away N-retinylidene-phosphatidylethanolamine (N-ret-PE), a byproduct of the visual cycle [11,12,13]. Failure of this flippase results in the accumulation of toxic byproducts. In patients, the accumulation is seen as debris at the photoreceptor-RPE interface accompanied by dystrophy and loss of photoreceptor and RPE cells. Vision loss ensues.

**Figure 2:**
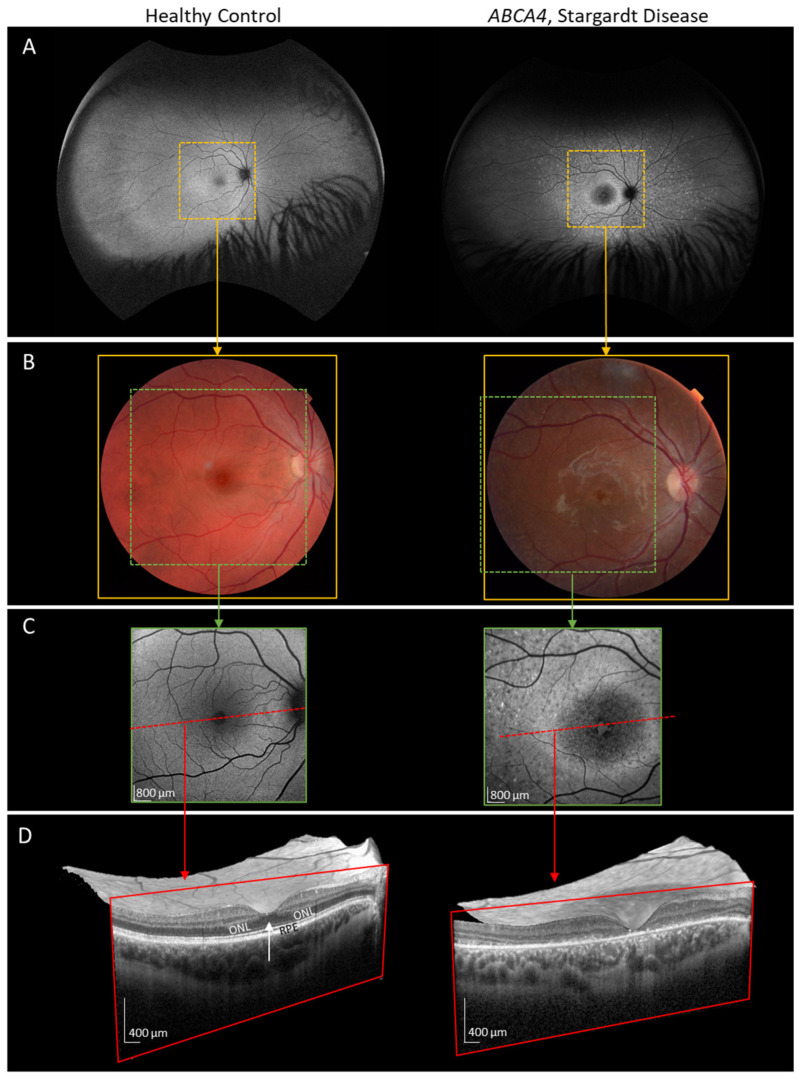
Retinal images of right eye. Left column: healthy Control 1. Right column: Patient 1. (**A**) Fundus autofluorescence (200°; California; Optos, Dunfermline, Scotland, UK); (**B**) color fundus photograph (45°; TRC-NW8F; Topcon Corporation, Tokyo, Japan); (**C**) blue autofluorescent image (30°; HRA + OCT Spectralis; Heidelberg Engineering, Heidelberg, Germany); (**D**) horizontal OCT showing the 30/61 b-scan slice (30°; HRA + OCT Spectralis; Heidelberg Engineering, Heidelberg, Germany). The dotted box in A indicates the region shown in (**B**). The dotted box in (**B**) indicates the region imaged in (**C**). The box in (**C**) indicates the region shown in (**D**). In (**C**), the dashed red line indicates the site of the OCT slice, as shown in (**D**). In the control, the OCT slice, bound by the red rectangle, has a dark band, the outer nuclear layer (ONL), representing photoreceptor nuclei; the ONL normally widens in subfoveal retina (white arrow). In the patient with STGD1, the ONL is absent in subfoveal retina, and there is debris at the retina–pigment epithelium (RPE) interface.

In Figure 2, we show the retinal images of healthy Control 1 (Table 1) side by side with those of Patient 1 (Table 1), who has typical features of STGD1, namely central macular atrophy with photoreceptors and RPE missing at the center of the macula. In the OCT images of the patient’s subfoveal and adjacent retina (red box), the photoreceptors have disappeared, and debris, known to be composed of bisretinoids associated with RPE lipofuscin, has accumulated [17,18].

**Figure 3:**
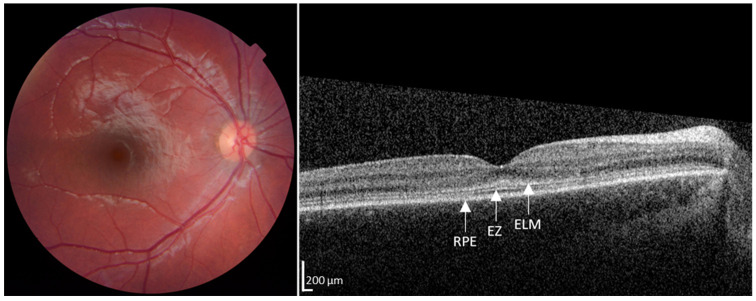
Fundus photograph and horizontal transfoveal OCT image of asymptomatic patient (Patient 2, Table 1) with biallelic pathogenic changes in *ABCA4*. The photograph (**left panel**), as well as ophthalmoscopy, showed no signs of maculopathy. The OCT (**right panel**) shows thickened and hyperreflective ELM and indistinct EZ similar to that reported by others [19,20].

Antedating central macular atrophy and the patient’s progression to legal blindness, as well as early changes in the fine structure of the photoreceptors, have been reported [19,20]. In OCTs [21,22], photoreceptor abnormalities, namely thickened and hyperreflective external limiting membrane (ELM) and an abnormal reflective signal from the ellipsoid zone (EZ), are early changes in photoreceptor structure. This is illustrated by images (Figure 3) from one of our young patients. Even if the central macula is atrophic, as shown in Figure 2, some patients’ OCTs have identifiable ELM, EZ, and RPE at other macular sites. We decided to analyze the structure of surviving STGD1 photoreceptors. We considered this a step toward identifying pathobiological processes in STGD1 photoreceptors other than the failure of the flippase [19,20].

Herein, we report STGD1 patients whose maculas included regions with relatively well-preserved photoreceptors having identifiable hyperreflective OCT bands [23]. These bands are the ELM, the optical correlate of the adherens junctions between the photoreceptors and glial cells of Muller, the EZ with abundant mitochondria, and the RPE.

It is established that experimental thinning of the outer retina from ELM to RPE (ELM-RPE) is mediated by pH-triggered water removal across the RPE. If the subretinal space is acidified due to dystrophy and oxidative stress, contraction of the ELM-RPE occurs [24,25,26,27,28,29]. Additionally, the mitochondrial configuration within photoreceptors (MCP) can be assessed by the aspect ratio (AR) of the intensity profile of the EZ. The MCP/AR, captured in OCT images, is responsive to bicarbonate, pH, and respiratory efficiency of the photoreceptors’ mitochondria [30,31,32]. Both ELM-RPE thickness and MCP/AR have been validated against gold-standard assays as biomarkers of mitochondrial activity and respiratory efficiency [27,30,31,32,33,34,35].

We used AO imaging [36,37,38] to characterize the photoreceptors in STGD1. AO imaging noninvasively enhances the resolution of the SLO [39,40,41] and the OCT [42] by correcting optical aberrations in the eye. This enables cellular-level resolution approaching the fidelity of light microscopy and thus allows the quantification of changes in cells induced by retinal disease in the living eye. We sampled cone distributions and inspected the photoreceptor-RPE interface [41,43,44] in STGD1 maculas, for which we also delineated MCP/AR.

Our long-term aim to preserve vision in our patients strongly motivates us to specify further the pathobiology of surviving STGD1 photoreceptors. We seek photoreceptor targets that are amenable to early intervention. Accordingly, we analyze the ELM-RPE and the MCP/AR [45,46] in relatively well-preserved photoreceptors. We exploit AO imaging to conduct cone counts and examine the RPE-photoreceptor interface [47,48,49,50].

## 2. Materials and Methods

### 2.1. Participants

We present five patients with genetically confirmed STGD1 (Table 1). The locations of the protein impacted by these variants are shown in Figure 1B. The severity of the biallelic genotype ranged from effectively double null (Patients 2 and 3), that is, producing no functional protein, to variants producing protein of less than half the normal functionality (Patients 1 and 4) to only mildly degraded protein functionality (Patient 5). For the discussion of the variants and genotypes, please see Appendix A. Patient 4 had a variant newly characterized in the Molday laboratory; these results are summarized in Appendix B.

We imaged three healthy controls (Table 1). For age and sex matching, we recruited Controls 2 and 3, specifically for the ELM-RPE and MCP/AR studies. Control 1 along with Patients 4 and 5 were recruited for AO-SLO and OCT imaging and coherence measurement of axial lengths (IOLMaster 500; Carl Zeiss Meditec, Jena, Germany). These participants, or the parents in the case of minors, provided written informed consent after explanation of the nature, purpose, and possible consequences of the procedures.

Best-Corrected Visual Acuity (BCVA) was measured during the course of clinical care at Boston Children’s Hospital. Spherical equivalent was derived from the results of cycloplegic refraction. Fundus photographs (TRC-NW8F; Topcon Corporation, Tokyo, Japan), wide-field images (California; Optos, Dunfermline, Scotland, UK), and SD-OCT (HRA + OCT Spectralis; Heidelberg Engineering, Heidelberg, Germany) have also been collected at clinical visits.

This study conformed to the tenets of the Declaration of Helsinki. It was approved by the Boston Children’s Hospital Institutional Review Board.

### 2.2. OCT Imaging

To collect the Spectralis OCT images, we dilated the pupil using a combination drop: Cyclopentolate 1%, Tropicamide 1%, and Phenylephrine 2.5% (Leiters, Englewood, CO, USA). Before imaging, we set the Spectralis device to IR and adjusted the focus to match the patient’s spherical equivalent. After making minor focus adjustments to optimize the image clarity, we centered horizontal B-scans (ART = 100, 30° scan angle) on the fovea. For the ELM-RPE and MCP/AR analysis [46], we chose horizontal transfoveal images with well-defined hyperreflective bands and as little tilt as possible.

### 2.3. Preparing the OCT Images for Analysis

**Figure 4:**
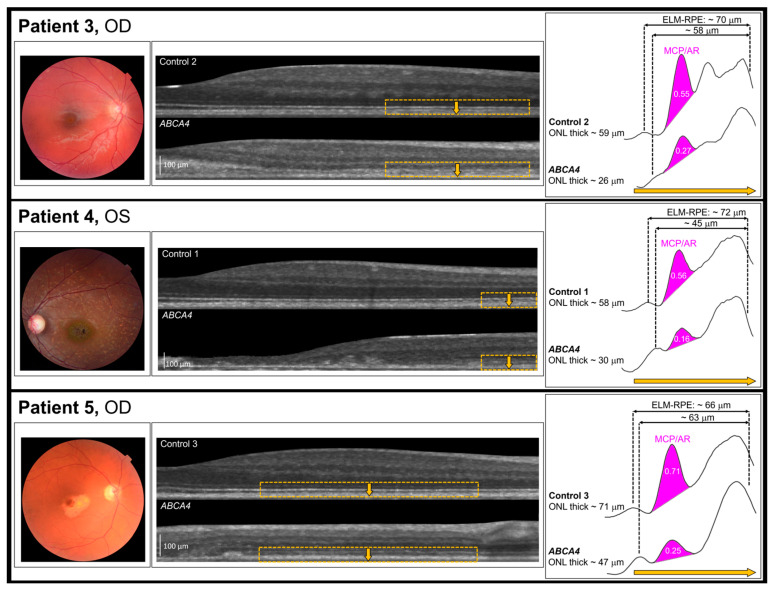
For each of the 3 patients (Patient 3, Patient 4, & Patient 5), from left to right we present, Fundus photograph, Flattened OCT images of retina nasal to the fovea for STGD1 and age- and sex- matched controls (See Table 1); the yellow boxes indicate the region of interest (ROI). The yellow arrow indicates the direction of the A-scan. In the right most panel the mitochondrial configuration within photoreceptors/aspect ratio (MCP/AR). Patient characteristics are shown in Table 1.

We imported minimally processed Spectralis database (SDB) files into ImageJ version 1.49m [51]. Pixel values are directly proportional to log-transformed reflectivities. We marked the location of the fovea and manually estimated the laminar boundaries for segmentation, including the hyperreflective band belonging to the RPE. We used previously developed R script version 3.3.1 [45,46,52] to refine these boundaries according to local peak reflectivity. We then resampled the retina along lines perpendicular to the RPE. This resampling generated linearized images, as shown below in Figure 4. The R script also generated the outer nuclear layer (ONL) thickness and ELM-RPE thickness. To measure MCP/AR from the OCT reflectance profile, we used R, MATLAB (R2021a; MathWorks, Natick, MA, USA) code, and ImageJ’s macros.

ELM-RPE and MCP/AR were then measured in a rectangular region of interest (ROI). To be included, we required identifiable ONL, ELM, EZ, and RPE. In the STGD1 eyes, the ROI was eccentric to the region of macular atrophy. In the control eyes, the ROI was eccentric to the fovea.

We then generated an average A-line reflectance profile reminiscent of the profile in Figures 6 and 7 of the classic paper of Spaide and Curcio [23]. We calculated ONL and ELM-RPE thicknesses and MCP/AR. As previously described, we set the MCP/AR baseline by connecting the local minima on either side. Finally, we used an ImageJ macro set for “shape descriptor”, “fit ellipse”, and “invert Y coordinates” to measure the minor and major axes of the AR. This process yields MCP/AR as a single value [45,46,53]. For more details, see the ImageJ source [53,54]. Note that in experimental studies, changes in the AR were independent of retinal hydration as measured by ELM-RPE thickness [45].

### 2.4. AO Imaging

Our multimodal AO retinal imager (MAORI; Physical Sciences, Inc., Andover, MA, USA) has been previously described in detail [47,48]. Cellular-level resolution is provided by AO correction in real time when scanning laser ophthalmoscopic (SLO) and OCT images. SLO and OCT images are acquired simultaneously and perfectly registered. Broadband (40–60 nm) light is used for illumination in the SLO (at 760 nm) and OCT (at 850 nm) channels. Both AO-SLO and AO-OCT images exhibit a lateral resolution of ~2.5 µm in a normal human eye, given a 7.5 mm beam diameter at the pupil. SLO detection includes confocal and offset channels [55,56,57]. In our imaging protocols, 64 images were acquired at a frame rate of 28 Hz, aligned, and registered using a non-rigid registration procedure [49]. Previous investigations of retinal diseases based on MAORI imaging have been reported [48,50].

We used a custom MATLAB code to identify local maxima. We obtained images using both confocal and offset apertures. Our expert graders [M.M. (PSI) and J.D.A.] manually added or removed marks for correct identification of cones.

## 3. Results

In STGD1, compared to controls, the ONL (optical correlate of photoreceptor nuclei) was thinner, and the distance from the ELM to RPE was shorter. In every STGD1 patient (Figure 4), MCP/AR was lower than in their control. Both cones and rods are normally present in the regions studied [58].

**Figure 5:**
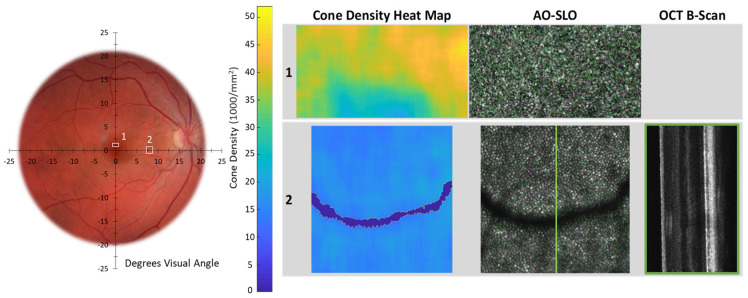
Healthy control 1. Far-left fundus photograph of a healthy control (right eye); location and size of AO Scan 1 and Scan 2 are as indicated. Left column—cone density heat maps. Center column—AO-SLO cone images with identified cones (green dots). Right column—OCT B-scan at the location indicated by the green line on the AO-SLO. AO imaging details are shown in Table 2.

**Figure 6:**
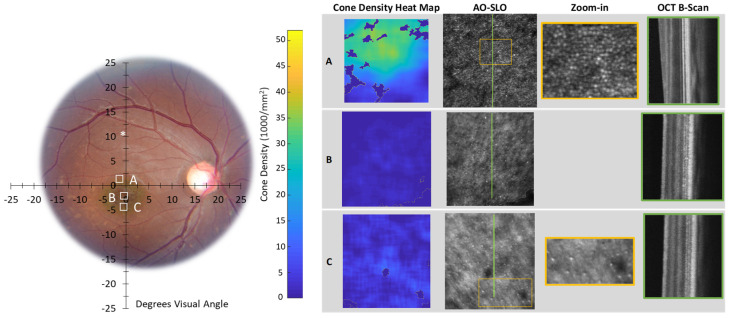
Patient 4, right eye. Format is similar to that of Figure 5, with the zoom-in (yellow square on the AO-SLO) column added. * in the superior retina, at ~10° is the patient’s preferred retinal locus for fixation (PRL). AO imaging details are shown in Table 2.

**Figure 7:**
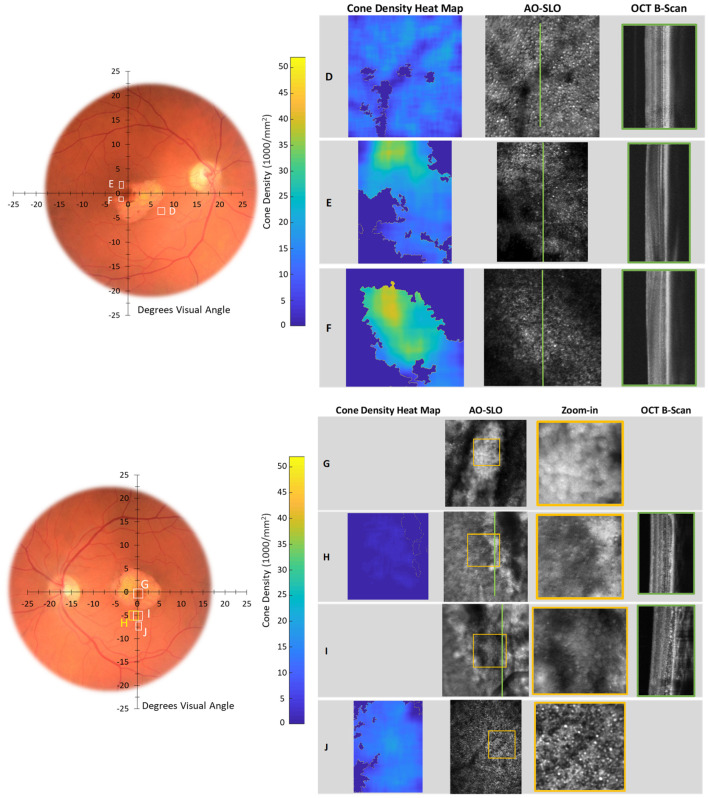
Patient 5, both eyes. Top-right eye scan (**D**–**F**). Bottom-left eye scan (**G**–**J**). Format is similar to that of Figure 6. AO imaging details are shown in Table 2.

In Table 2, we summarize our AO imaging results and compare the cone counts in our participants to the range reported for normal retina [58]. Figure 5, Figure 6 and Figure 7 show the AO images. We marked the accompanying fundus photograph to indicate the site and size of the AO-SLO scans. Where available, we also show the AO-OCT B-scans. As mentioned above, having pushed the focal plane down to the photoreceptor layer, we obtained, in the register, simultaneous SLO and OCT images. The heat maps show the local spatial distribution of cones; bright yellow indicates regions of high density, and dark blue indicates no cones identified. On the AO-SLO, we identified and marked the cones, as illustrated in Figure 5. Where available, we show the companion vertical AO-OCT B-scans. In Appendix C, we present overlaps of Topcon fundus photographs and Spectralis SLO and OCT images for Control 1 (A2), Patient 4 (A3), and Patient 5 (A4), along with fixation plots obtained using the macular integrity assessment microperimeter (MAIA; CenterVue, Padova, Italy).

In Figure 5, we show Scan 1 and Scan 2 of a healthy control retina. Cone counts meet or exceed the reported normal range at 1° and 10° eccentric. The heat maps summarize the distribution of cones in the regions studied. Blue-green shades in Scan 1 are the result of insufficient image resolution due to the limited AO correction. Cones are not well resolved in the fovea where there is high cone density. The spacing between the foveal cones is smaller than the lateral resolution of the system.

Corresponding data from Patients 4 and 5 (Figure 6 and Figure 7) show non-uniform cone distributions. For Patient 4, zoom-ins (yellow boxes) show magnified views to better visualize the lesion structure and the cone mosaic. In Patient 4, at the edge of the macular lesion (Figure 6, Scan A), there is an area with normal cone density (greenish on the heat map). Within the lesion are areas in which cones are very sparse, well below the normal range (Figure 6, Scans B and C; Table 2).

For Patient 5 (Figure 7; Table 2), cone densities varied from those greater than normal to unambiguously sparse in both right (scans D, E, F) and left (scans G, H, I, J) maculas. For the right eye, scans E and F at the edge of the lesion show areas with normal cone densities (yellow-green shades) and other areas where cones are sparse (blue shades). Away from the lesion, ~8° eccentric cone densities exceed the normal range, suggesting a redistribution of the cells in this maculopathy.

For the left eye of Patient 5, higher than normal cone density is found eccentric to the fovea (scan J), while closer to the lesion (scans H, I), cones are sparse or absent. At the lesion (scan G), no cones are seen. The AO-OCT, registered with scans H and I, shows significant thinning of the photoreceptor layer and accumulated debris at the photoreceptor-RPE.

Some regions of the AO-SLO images had low intensity. These regions, indicated by the dark geographic patches within the heat maps, contained no cones or very few cones—below a threshold of 1000 cones/mm^2^—and were not included in the calculation of the mean cone density. The low intensity in these regions could be due to several reasons: (a) no cones capable of reflecting light in the confocal images, (b) a shadow cast by capillaries located above the imaged area which basically obscures the cones below them, or (c) intraretinal deposits which can also hide the photoreceptors.

## 4. Discussion

In this study of surviving, but not thriving, photoreceptors in STGD1, we characterized abnormalities in the fine structure of the inner segment (Figure 4) and documented cone density from very sparse to greater than normal (Figure 5, Figure 6 and Figure 7; Table 2). These appear to be common features across our patients. Despite an unabashedly small sample, our patients cover a range of ages and clinical presentations. The severity of their *ABCA4* variants includes those incapable of producing any protein. Other genetic variants do produce protein, but the protein is abnormal. The abnormal protein is located at different sites in the photoreceptor outer segment: in the disc membrane, in the disc lumen, or within the cytosol (Figure 1B; Appendix A). We have sampled regions of the macula in which cones normally outnumber rods and, at the greatest eccentricities (7°, 8°, 10°), where rods outnumber cones [58]. The non-uniform distribution of cones in STGD1 has been demonstrated in a number of previous AO imaging studies [19,59,60,61].

In the surviving photoreceptors, we find OCT evidence of inner segment abnormalities (Figure 4). These pilot data for the MCP/AR in STGD1 sparked our interest in the role of mitochondria in the STGD1 photoreceptors. First, in this progressive dystrophy, the STGD1 photoreceptor’s metabolic machinery may operate in a milieu of decreased (acidified) pH in the subretinal space accompanied by contraction of the ELM-RPE [27,29,33,35,62,63]. Such may be the consequence of some combination of microglia activation, oxidative stress, and other processes linked to dystrophy [45,64]. Second, the surviving photoreceptors must prevail, as best as possible, to balance ATP production and use. Laboratory perturbations that create a putative alkaline milieu impact the MCP/AR and distribution of the mitochondria [46]. We are also reminded of laboratory studies that show mitochondria are associated with complex cellular responses [65,66], thus raising the possibility of an array of therapeutic approaches [64].

The AO studies (Figure 6 and Figure 7) show the distribution of surviving STGD1 photoreceptors ranges from normal to unambiguously sparse. In Patient 4 and Patient 5, we used AO imaging to sample macular regions similar to those included in the MCP/AR analyses. We used both modalities, AO imaging and MCP/AR analyses, to study similar macular regions in Patient 4 and Patient 5. We found abnormal MCP/AR in regions with cone densities and debris-free photoreceptor-RPE interfaces that are indistinguishable from normal. Thus, the MCP/AR abnormalities (Figure 4) may represent an early phase of the STGD1 photoreceptor’s disease.

Where cone densities are lower than normal, there is debris at the photoreceptor-RPE interface, a hallmark of failed flippase and severe photoreceptor disease. A goal of therapies would be to protect the surviving photoreceptors and RPE from further dystrophy, which is the natural course of STGD1 maculopathy. Fortunately, our study and those of others indicated that surviving STGD1 photoreceptors are not rare [19,59,60,61], and the data summarized in Figure 4 lead to suggestions for early pharmaceutical intervention.

## 5. Conclusions

Some macular regions in the patients with STGD1 contain persistent but abnormal photoreceptors. Through the study of the optical correlates of the metabolic machinery in these surviving photoreceptors, we are led to consider a pharmaceutical approach to protect these photoreceptors. A real-world challenge remains how to identify patients with STGD1 before any vision loss. With the development of genetic screening programs, there is hope that such can pertain to Stargardt disease.

## Figures and Tables

**Table 1:** Genetic and clinical characteristics of patients with STGD1 and controls.

Participant		Age at Imaging (Years)		Sex	Allele 1 (cDNA, Protein)	Allele 2 (cDNA, Protein)	BCVA (logMAR) OD, OS	Spherical Equivalent (Diopter) OD, OS	Axial Length (mm) OD, OS
		OCT	AO						
Patients	1	15	-	F	c.4036_4037del (p.T1346Gfs*75)	c.6112C>T (p.R2038W) c. 6569A>G (p.Q2190R)	0.875, 0.796	−0.75, −0.75	-
	2	3	-	M	c.4469G>A (p.C1490Y)	c.5461-10T>C (splice)	0.301, 0.301	Plano, plano	-
	3	5	-	F	c.4469G>A (p.C1490Y)	c.5461-10T>C (splice)	0.176, 0.097	1.00, 1.00	-
	4	25	17	F	c.2966T>C (p.V989A)	c.6286G>A (p.E2096K)	0.602, 0.602	−0.50, −0.50	23.40, 23.18
	5	61	58	M	c.2588G>C (p.G863A)	c.5882G>A (p.G1961E)	0.000, 0.176	−3.50, −3.13	25.06, 24.84
Controls	1	25	24	F	-	-	−0.04, −0.10	−0.38, −1.75	24.52, -
	2	5	-	F	-	-	0.18 0.18	+4.50, +4.36	-
	3	61	-	M	-	-	0.00, 0.00	-	-

**Table 2:** AO imaging details.

Participant	Scan	Eye	Aperture	Scan Size		Zoom-in		Eccentricity		Cone Density [1000/mm^2^]			Figure Number(s) in This Paper
				[°]	[µm × µm]	[°]	[µm × µm]	[°]	[mm]	Mean	Max	Normal Range [58]	
Control 1	1	OD	confocal	1.4 × 0.9	420 × 260			1	0.3	39	52	50–52	Figure 5 and Figure A2
	2	OD	confocal	1.4 × 1.4	420 × 420			10	3.0	17	24	8–9	
Patient 4	A	OD	confocal	1.1 × 1.0	310 × 280	0.40 × 0.30	100 × 75	2	0.6	19	38	30–50	Figure 6 and Figure A3
	B	OD	offset	1.0 × 1.0	280 × 280	-	-	3	0.7	3	14	25–40	
	C	OD	offset	1.0 × 1.0	280 × 280	0.60 × 0.30	170 × 84	4	1.1	4	9	12–25	
Patient 5	D	OD	offset	1.0 × 1.0	310 × 310	-	-	8	2.5	11	25	8–9	Figure 7 and Figure A4
	E	OD	confocal	0.9 × 1.1	280 × 340	-	-	1	0.4	14	39	30–40	
	F	OD	confocal	0.9 × 0.8	280 × 250	-	-	1	0.4	16	45	30–40	
	G	OS	offset	2.0 × 2.0	610 × 610	0.60 × 0.60	180 × 180	~0	-	-	-	-	
	H	OS	offset	2.0 × 2.0	610 × 610	0.80 × 0.80	230 × 230	5	1.5	2	4	12–13	
	I	OS	offset	2.0 × 2.0	610 × 610	0.80 × 0.80	230 × 230	5	1.5	-	-	-	
	J	OS	confocal	1.3 × 1.6	400 × 490	0.50 × 0.50	150 × 150	7	2.1	11	22	9–10	

**Figure A1:**
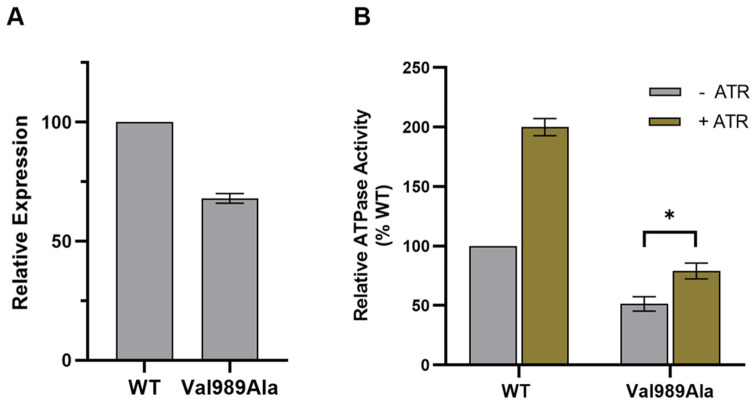
Biochemical characterization of the Val989Ala variant associated with Stargardt disease. WT *ABCA4* and the Val989Ala variant were expressed in HEK293T cells. (**A**) Protein expression levels were determined on Western blots labeled for *ABCA4*. The Val989Ala expression was 70% that of WT *ABCA4*. (**B**) The ATPase activity of purified WT and the Val989Ala variant was determined at the same protein concentration in the absence (−) and presence (+) of all-trans-retinal (ATR). Data are expressed relative to WT *ABCA4* in the absence of ATR. A small but significant (*p* < 0.05) activation by ATR was observed for the Val989Ala variant. Data show SD for *n* = 3 independent experiments.

**Figure A2:**
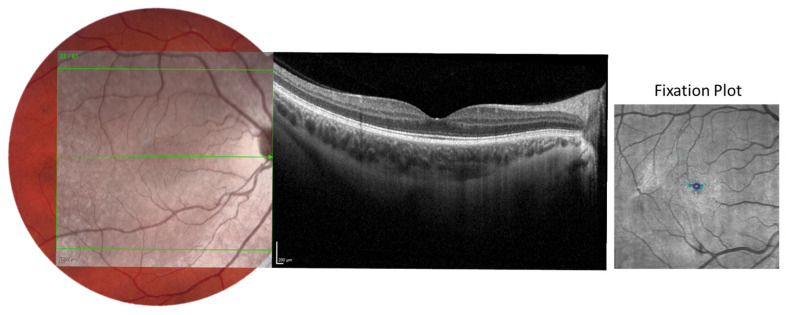
Fovea OCT image from the right eye from a healthy normal control overlaid on their fundus photo. Note the intact layers of the retina and how their fixation in the fixation plot is centered in their foveola, and the Bivariate Contour Ellipse (BCEA) is small (63% BCEA: 0.4° × 0.3°, area = 0.1°^2^, angle = −6.5°).

**Figure A3:**
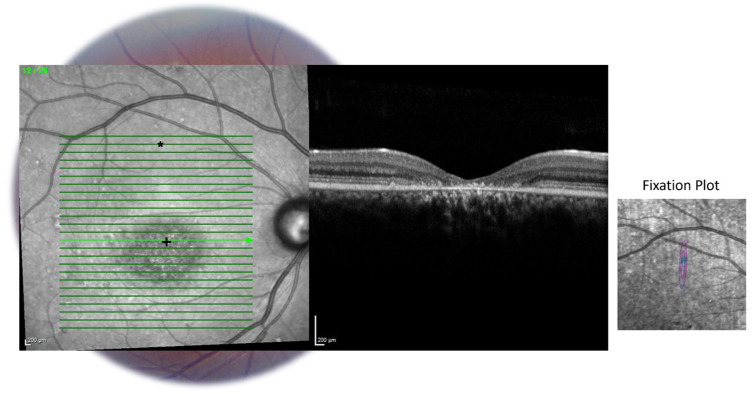
Fovea OCT image from the right eye from Patient 4 overlaid on fundus photo at age 16.8 years old. * marks the patient’s fixation during adaptive optics testing session. The fixation plot taken from their MAIA exam at 25 years confirms this fixation location (63% BCEA: 0.5° × 3.9°, area = 1.7°^2^, angle = 87.3°). + on the fundus marks the fovea as shown in OCT slice.

**Figure A4:**
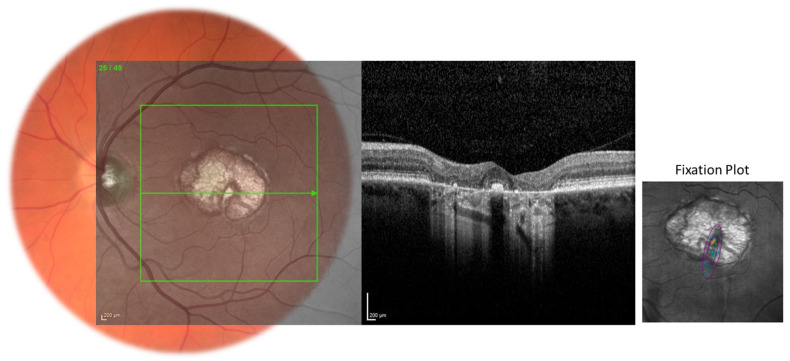
Fovea OCT image from the left eye from Patient 5 overlaid on fundus photo at age 58 years old. The fixation plot taken from their MAIA exam at 60 years confirms this fixation location (63% BCEA: 1.2° × 4.1°, area = 3.8°^2^, angle = 77.7°). Note how the patient is able to fixate centrally due to foveal sparing.

## Data Availability

The data presented in this study are available on request from the corresponding author. The data are not publicly available due to protected patient information.

## Appendix A

Patient 1 is a carrier of one putative null allele, c.4036_4037del, p.Thr1346GlyfsTer75, leading to an early stop codon. This presumably leads to the loss of mRNA through the nonsense-mediated decay (NMD) mechanism [67]. The other allele contains two variants. The variant c.6112C>T, p.Arg2038Trp was shown by Biswas-Fiss et al. [68] to decrease ATPase function by more than 60%. The other variant, c.6569A>G, p.Gln2190Arg, is, as of this writing, not experimentally characterized. While this genotype does not result in the complete loss of functional protein, its expression level is probably well below 40% of the wild type (WT).

The early onset of disease in Patients 2 and 3 is consistent with their genotype being a pair of alleles, neither of which produce any functional protein. The intronic variant c.5461-10T>C has been reported as the third most common *ABCA4* variant associated with Stargardt disease in patients of European and African descent [69]. As a noncanonical splice site variant, it causes splicing defects. Using photoreceptor progenitor cells, Sangermano et al. [70] showed that this variant leads to skipping of either exon 39 or of exon 39 and 40 in mRNA, resulting in a reading frameshift, an early stop in codon 10, and presumably loss of protein product through NMD. Aukrust et al. [71] confirmed these findings using fibroblast samples from patients with STGD1. Low levels of normally spliced full-length *ABCA4* mRNA and non-truncated, functional *ABCA4* protein were detected in hetero- and homozygotes [71].

The missense variant c.4469G>A (p.C1490Y), located in extracellular domain 2 (ECD2; Figure 1), was characterized by Schulz et al. [72]. Interestingly, this variant not only reduces substrate-stimulated ATPase activity (possibly due to impaired retinal binding [73]), but it also creates a cryptic donor splicing site in exon 30. This variant results in the loss of part of exon 30 in the mRNA product and leads to an early stop codon and putative loss of protein product through NMD [72].

Patient 4 is a carrier of two missense variants. The missense variant c.6286G>A (p.Glu2096Lys) results in the substitution of the conserved glutamate residue in the Walker B motif hhhhAspGlu (where h is a hydrophobic residue) of NBD2 required for ATPase hydrolysis with a positively-charged lysine residue. This substitution does not significantly affect protein expression but reduces basal ATPase activity by 50% (the remaining basal ATPase activity is from NBD1) and completely abolishes substrate-stimulated ATPase activity [73].

The missense variant c.2966T>C (p.Val989Ala) results in the replacement of an amino acid having a bulky, non-polar side chain in NBD1 with one smaller nonpolar side chain. In this case, the expression of the variant is reduced to 70% of the normal WT expression, and some substrate-activated ATPase activity is retained (Figure A1). Thus, the combined effect of these two alleles is this: more than half of the *ABCA4* protein that finds its way into the membrane is functionally inactive, and the remaining fraction works at diminished capacity. The details of this newly characterized variant are presented in Appendix B.

Patient 5 carries two well-characterized missense variants. Consistent with the late onset in this patient, both variants result in an expressed protein with only partially degraded function. Missense variant c.5882G>A (p.Gly1961Glu) was comprehensively characterized by Garces et al. [69]. It is the most common pathogenic *ABCA4* variant located in NBD2. While the protein expression, folding, and membrane embedding are similar to that of the healthy geneWT, N-Ret-PE binding is significantly diminished, both in the absence and in the presence of ATP. The ATPase activity of CHAPS detergent-solubilized Gly1961Glu is significantly reduced in both basal and retinal-stimulated conditions. While in vitro studies show that this variant causes severe *ABCA4* dysfunction, clinical data point to a milder, late-onset phenotype. This discrepancy might be a result of experimental conditions, as the CHAPS detergent used for solubilization and purification of this variant might cause irreversible denaturation of this specific variant and severe loss of function [69].

Missense variant c.2588G>C (p.Gly863Ala) is located in TMD1, close to NBD1. Functional characterization by Curtis et al. [74] showed that this variant is expressed but at levels modestly lower than in the WT. Basal and retinal-activated ATPase activities are comparable to WT, as is the ability to bind N-Ret-PE, both in the presence and the absence of ATP. This variant has been characterized as hypomorphic because it does not significantly diminish *ABCA4* function and is associated with a later age of symptom onset [74].
